# Molecular dissection of heterosis in cereal roots and their rhizosphere

**DOI:** 10.1007/s00122-023-04419-6

**Published:** 2023-07-20

**Authors:** Jutta A. Baldauf, Frank Hochholdinger

**Affiliations:** Institute of Crop Science and Resource Conservation, Crop Functional Genomics, University of Bonn, 53113 Bonn, Germany

## Abstract

**Key message:**

Heterosis is already manifested early in root development. Consistent with the dominance model of heterosis, gene expression complementation is a general mechanism that contributes to phenotypic heterosis in maize hybrids.

**Abstract:**

Highly heterozygous F_1_-hybrids outperform their parental inbred lines, a phenomenon known as heterosis. Utilization of heterosis is of paramount agricultural importance and has been widely applied to increase yield in many crop cultivars. Plant roots display heterosis for many traits and are an important target for further crop improvement. To explain the molecular basis of heterosis, several genetic hypotheses have been proposed. In recent years, high-throughput gene expression profiling techniques have been applied to investigate hybrid vigor. Consistent with the classical genetic dominance model, gene expression complementation has been demonstrated to be a general mechanism to contribute to phenotypic heterosis in diverse maize hybrids. Functional classification of these genes supported the notion that gene expression complementation can dynamically promote hybrid vigor under fluctuating environmental conditions. Hybrids tend to respond differently to available nutrients in the soil. It was hypothesized that hybrid vigor is promoted through a higher nutrient use efficiency which is linked to an improved root system performance of hybrids in comparison to their inbred parents. Recently, the interaction between soil microbes and their plant host was added as further dimension to disentangle heterosis in the belowground part of plants. Soil microbes influenced the performance of maize hybrids as illustrated in comparisons of sterile soil and soil inhabited by beneficial microorganisms.

## Heterosis, an important agronomic phenomenon with a long history

Cross-fertilization of two distinct homozygous inbred lines results in a F_1_-hybrid with superior phenotypic performance when compared to its parents. This phenomenon was first described in tobacco by Joseph Kölreuter (Kölreuter 1761) and later by Charles Darwin in more than 60 plant species (Darwin 1876). “Heterosis”, a term which was introduced by George H. Shull for the observed hybrid vigor (Shull 1948), occurs in a variety of species and affects diverse morphological and physiological traits.

**Fig. 1:**
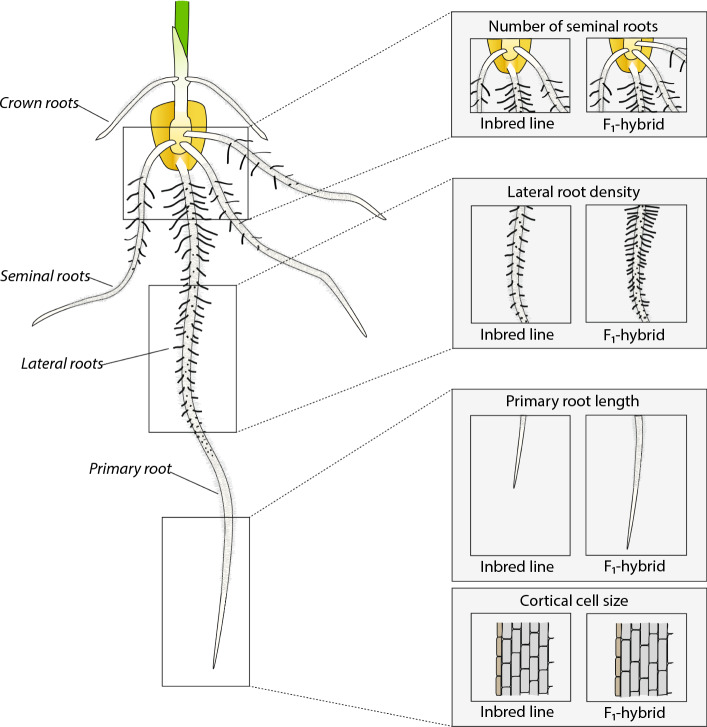
Maize seedling root system and associated traits displaying heterosis. Hybrids display in comparison to their parental inbred lines an increased number of seminal roots, a higher density of lateral roots, a longer primary root and an increased cortical cell size. According to data from (Hoecker et al. 2006)

In general, heterozygous F_1_-hybrids exhibit increased yield and biomass (reviewed in Hochholdinger and Baldauf 2018) and display an improved phenotypic stability under abiotic or biotic stress conditions (Fridman 2015) than their homozygous parental inbred lines. Although hybrid vigor is mainly observed for yield related traits late in development, heterosis is already manifested during embryo development (Meyer et al. 2007) and early seedling development (Chairi et al. 2016). Hence, it can also be monitored for numerous traits in young seedling roots (Hoecker et al. 2006; Fig. 1). Importantly, the level of heterosis can vary significantly between different traits across an individual plant (Paschold et al. 2010).

The degree of heterosis depends on the genetic composition of the parental inbred lines and the pollination type of the species. Hybrids of maize (*Zea mays* L.), as a typical outcrossing species, achieve for instance 50–100% yield increase in comparison to open-pollinated maize varieties (Kutka 2011). In contrast, hybrids of the naturally self-pollinating species rice (*Oryza sativa* L.) display only a yield increase of 10–20% in comparison to their corresponding inbred line varieties (Huang et al. 2017). Plant breeders assorted the germplasms into diverse heterotic groups, since it was recognized that inter-group hybrids reveal stronger heterosis than intra-group hybrids in maize (Melchinger and Gumber 1998). These groups experienced several rounds of genetic and morphological changes, especially with respect to agronomically important traits, to produce more promising inbred lines and subsequently higher yielding hybrids (Li et al. 2022).

## Utilization of heterosis in agriculture

**Fig. 2:**
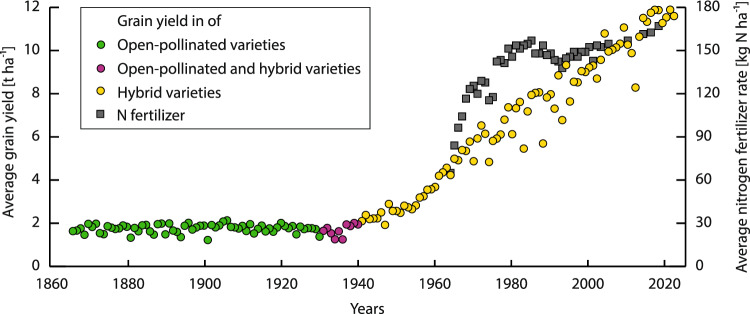
Average grain yield in t ha^.1^ of maize varieties and average nitrogen (N) fertilizer application rate in kg N ha^−1^ for maize production in the USA. Yield of open-pollinated varieties (green) from 1866 to 1930, of open-pollinated and hybrid varieties (magenta) from 1931 to 1940, and of hybrid varieties (yellow) from 1941 to 2022. Rate of N fertilizer application (gray) documented from 1964 to 2018. Data source: USDA-ERS 2022a, 2022b

It is projected by the United Nations that the human population will increase up to 10 billion by 2050 (Adam 2021), entailing optimized agricultural practices and crop improvement to secure global food production in a sustainable way. Hybrid breeding can contribute to increase food production. Since the introduction of the first commercial maize hybrid in 1930, grain yields increased by more than six-fold (Fig. 2; reviewed in Duvick 2005; Hochholdinger and Baldauf 2018). In parallel to the exploitation of hybrid vigor in cereals, there was also a rapid progress in the release of hybrid varieties in horticultural crops with the first commercial hybrid eggplant already introduced in 1924 (reviewed in: Yu et al. 2021a). Hybrid breeding is applied to around 50 of ca. 200 crop species with substantial agricultural importance (ter Steeg et al. 2022). For many crops, hybrids are often the first choice of farmers because of their uniformity and higher yields, which is reflected by a substantial market share of these varieties. In the Northern hemisphere, the commercial cultivation of maize is, for example, almost exclusively hybrid-based (reviewed in: Hochholdinger and Baldauf 2018).

## Belowground manifestation of heterosis

The effect of heterosis is typically recorded for aboveground traits in mature plants. However, considerable heterotic effects were also observed for diverse root traits in a number of species and at all stages of development (Fig. 1, Table 1), ranging from seedling to tillering and heading stage (Wang et al. 2006; Hoecker et al. 2006; Sharma et al. 2010; Hund et al. 2012; Zhai et al. 2013; Chairi et al. 2016; Kamphorst et al. 2022). Plant roots and their spatial arrangement in the soil play important roles in supporting plant growth and directly influence plant performance and yield (Hochholdinger 2009; Rogers and Benfey 2015). In many model and crop species the importance of root research for increasing overall plant productivity has been highlighted (Rogers and Benfey 2015; Hochholdinger 2016; Amtmann et al. 2022). Heterosis studies mainly focus on variation in root system architecture, i.e., the spatial arrangement of the roots in soil, rather than root function and its effect on plant performance (Table 1). Recently, a new perspective on root-mediated yield heterosis was provided by characterizing a common melon variety grafted onto 190 diverse hybrid rootstocks. The use of hybrid rootstocks resulted in a 40% yield increase compared to the parents (Dafna et al. 2021).

**Table 1:** Heterosis manifestation in diverse root traits of different cereals

Species	Root trait	MPH [%]	Developmental stage	References
*Zea mays L.*	Primary root length	17 to 26	Seedling stage [3–7 DAG]	Hoecker et al. (2006)
	Primary root width	1 to 7		
	Cortical cell size	24		
	Lateral root density	51		
	Seminal root number	18		
	Root weight density	90	6–8 Leaf stage	Chairi et al. (2016)
	Root length density	21		
	Specific root length	− 34		
	Root/shoot ratio	− 4		
	Primary root length	10		
	Seminal root length	83		
	Brace root length	15		
	Crown root length	− 48		
	Dry weight of primary roots	100		
	Dry weight of seminal roots	95		
	Dry weight of crown roots	109		
	Dry weight of brace roots	− 14		
	Root weight density	61 to 85	V13 [30 DAG]	Kamphorst et al. (2022)
	Specific root length	− 37 to − 26		
	Shoot/root ratio	− 22		
*Triticum aestivum L.*	Total root length	47	Jointing stage	Wang et al. (2006)
	Root surface area	54		
	Root average diameter	71		
	No. of root tips	33		
	Longest root length	20		
	Root volume	47		
	Root/shoot ratio	7		
	Root biomass	37		
	Root biomass	34	Tillering stage [45 DAG]	Sharma et al. (2010)
*Oryza sativa L*	Root length	16	Tillering stage	Zhai et al. (2013)
	Root dry weight	69		
	Root/shoot ratio	28		
	Root length	50	Heading stage	
	Root dry weight	137		
	Root/shoot ratio	29		

*MPH*  mid-parent heterosis; *DAG*  days after germination

## Genetic and molecular hypotheses to explain heterosis

Heterosis is a complex phenomenon caused by the combination of many factors. Classically, three non-exclusive genetic mechanisms have been proposed to explain heterosis: dominance, overdominance and epistasis.

**Fig. 3:**
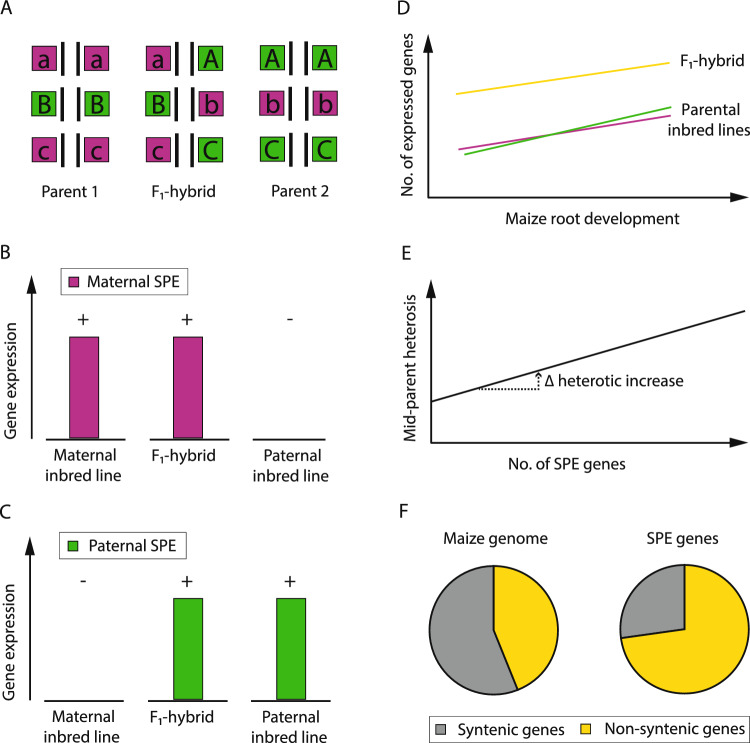
Single-parent expression complementation, a specific instance of the classical dominance model of heterosis. **A** Schematic depiction of the dominance model (modified according to Birchler 2006): In the F_1_-hybrid, for each gene (A, B, C), the deleterious parental allele (magenta) is complemented by the superior allele (green) of the second parental inbred line. **B** Maternally-derived (magenta) SPE pattern: Genes which are expressed (“ + ”) in the F_1_-hybrid and the maternal inbred line (magenta), but not in the paternal inbred line (“-”). **C** Paternally-derived (green) SPE pattern: Genes which are expressed (“ + ”) in the F_1_-hybrid and the paternal inbred line (green), but not in the maternal inbred line (“-”). **D** As a consequence of SPE complementation, hybrids (yellow) display a higher number of expressed genes than their parental inbred lines (magenta, green) along root development (modified according to Baldauf et al. 2018). **E** Phenotypic association of the number of SPE genes on mid-parent heterosis results in a heterotic increase. **F** Evolutionary origin of the modern maize genome (left) based on the proportion of syntenic genes (gray) and non-syntenic genes (yellow) and overrepresentation of non-syntenic SPE genes (right, yellow) compared to syntenic SPE genes (right, gray) (color figure online)

The dominance model, which was first reported by Charles Davenport and extended by Donald Jones, describes the concept that the two alleles of both parents are complemented in the hybrid and suppression of the recessive allele by the dominant one leads to the superior hybrid phenotype (Fig. 3A; Davenport 1908; Jones 1917). This model was supported by several studies observing an accumulation of multiple loci exhibiting dominance effects that contribute, for example, to grain yield heterosis in rice (Huang et al. 2015) or superior phosphate acquisition in an Arabidopsis hybrid (Narang and Altmann 2001).

Furthermore, the overdominance model suggests that the effect of heterosis relies on the interaction of heterozygous alleles in the hybrid, which is much stronger than between homozygous alleles (Shull 1908). In addition, the interaction of closely linked alleles can result in an overdominance effect that is known as pseudo-overdominance (Stuber et al. 1992). Like many complex quantitative traits, which are controlled by a network of genes, the dominance and overdominance hypothesis suggest both that heterosis is caused by the accumulation of various dominant or overdominant effects. However, there are some examples of single-locus overdominance, for example the *SINGLE FLOWER TRUSS (SFT*) gene, which mediates yield heterosis in tomato, or a very short root phenotype in wheat (Krieger et al. 2010; Li et al. 2013), but single gene heterosis is rare.

Finally, epistasis, i.e., interactions in the hybrid between non-allelic genes at one or more loci result in superiority, was proposed as an explanation for heterosis (Richey 1942; Powers 1944).

There are several examples supporting either hypothesis, but it is apparent that none of the classical genetic hypotheses can explain heterosis alone. Hence, some other models try to explain heterosis by an overall improvement in the energy utilization of the hybrid: in line with the gene balance hypothesis it was proposed that hybrids might have more favorable gene dosage balances than their parental inbred lines (Birchler et al. 2005; Birchler and Veitia 2007, 2010). A refinement of the hypothesis added an energy control component to the model (Goff 2010). According to this theory, hybrids are more energy efficient by selecting less cost-intensive protein biosynthesis and metabolism over energy-intensive processes via allele-specific expression (Goff 2010).

## Gene expression complementation is a general mechanism throughout hybrid development

From a molecular perspective, there is increasing evidence that variation of transcriptional regulation relates to the enhanced phenotypic performance of hybrids (Botet and Keurentjes 2020). Substantial research was performed to investigate heterosis manifestation at the level of transcriptomic differences between hybrids and their parental inbred lines, such as differential gene expression relative to the mid-parent level and allele-specific expression patterns (Guo et al. 2006; Springer and Stupar 2007; Paschold et al. 2012; Baldauf et al. 2016, 2020; Shao et al. 2019). Similarly, protein accumulation in roots of maize hybrids relative to their mid-parent value has been studied on the proteome level (Hoecker et al. 2008; Marcon et al. 2010, 2013; Rockenbach et al. 2018).

In a transcriptomic study of maize, an extreme instance of gene expression variation was observed in which hundreds of genes were expressed in the hybrids, but only in one of its two parental inbred lines: an expression pattern denoted as single-parent expression (SPE; Fig. 3B, C; Paschold et al. 2012). SPE genes are classified as maternally- or paternally-derived SPE genes depending on the expression of the gene in the maternal inbred line (maternal SPE; Fig. 3B), but not in the paternal, or vice versa (paternal SPE; Fig. 3C). The concept of expression complementation of the silent parental gene in the hybrid (Fig. 3B, C) is consistent with the classical model of dominance (Fig. 3A). Using a diverse panel of maize genotypes and different vegetative and generative tissues of seedling and mature maize plants, SPE complementation was demonstrated to be a general mechanism to increase the total number of active genes in hybrids (Fig. 3D; Paschold et al. 2014; Baldauf et al. 2018, 2022; Li et al. 2021b).

SPE complementation was proposed as a potential link to translate genetic distance of parental inbred lines into phenotypic heterosis. The number of identified SPE genes varies among genetically diverse hybrids (Table 2) but reflects the relatedness of the parental inbred lines (Baldauf et al. 2018, 2022; Li et al. 2021b). A similar pattern was observed for phenotypic mid-parent heterosis for diverse traits, which was significantly correlated to the number of identified SPE genes (Fig. 3E, Baldauf et al. 2022). Interestingly, the number of SPE genes determined in seedling primary roots were positively correlated to the degree of heterosis for traits measured at the adult stage of the plant. Although there is a link between expression complementation and heterosis, it was further demonstrated that specific groups of SPE genes affect the hybrid phenotype differently. In maize hybrids, with the inbred line B73 as maternal parent, the highly connected group of maternally-derived SPE genes were functionally associated with growth and development, whereas the group of paternally-derived SPE genes were highly associated with defense and stress response (Baldauf et al. 2022). In the same line, biological pathway expression complementation was recently observed in a hybrid cross between two Arabidopsis ecotypes (Liu et al. 2021). In comparison to its parental inbred lines, the hybrid exhibited spatio-temporal complementary dominant expression patterns for genes involved in cell division and photosynthesis during different stages of seedling development (Liu et al. 2021). These results provide further cues on expression complementation as a dynamic process to support hybrid vigor.

**Table 2:** Number of genes displaying single-parent expression (SPE) pattern in diverse maize genotypes and tissues

Hybrid genotype	Tissue/condition	Developmental stage	No. of SPE genes		Non-syntenic SPE genes [%]		References
			SPE_M	SPE_P	SPE_M	SPE_P	
B73xMo17 Mo17xB73	Primary root	Seedling stage [2–4 cm length]	358^1^	766^2^	NA	NA	Paschold et al. (2012)
	Cortex	Seedling stage [2–4 cm length]	599^1^	661^2^	75^5^	75^5^	Paschold et al. (2014)
	Stele		607^1^	680^2^	77^5^	77^5^	
	Meristematic zone		510^1^	581^2^	78^5^	78^5^	
	Elongation zone		472^1^	565^2^	82^5^	82^5^	
	Primary root/Control	Seedling stage [2–4 cm length]	853^1^	1144^2^	74^5^	74^5^	Marcon et al. (2017)
	Primary root/WD	Seedling stage [2–4 cm length]	841^1^	1183^2^	75^5^	75^5^	
B73xA554	Primary root	Seedling stage [2–4 cm length]	1025	979	60	66	Baldauf et al. (2018)
		Seedling stage [6–8 cm length]	1090	954	62	68	
		Seedling stage [10–12 cm length]	1135	920	63	71	
B73xH84	Primary root	Seedling stage [2–4 cm length]	996	874	62	70	
		Seedling stage [6–8 cm length]	916	852	65	71	Baldauf et al. (2018)
		Seedling stage [10–12 cm length]	966	808	61	72	
B73xH99	Primary root	Seedling stage [2–4 cm length]	1139	1001	63	65	Baldauf et al. (2018)
		Seedling stage [6–8 cm length]	1028	994	62	66	
		Seedling stage [10–12 cm length]	1072	935	64	69	
B73xMo17	Primary root	Seedling stage [2–4 cm length]	1183	1083	61	66	Baldauf et al. (2018)
		Seedling stage [6–8 cm length]	1113	951	64	68	
		Seedling stage [10–12 cm length]	1035	968	65	72	
B73xOh43	Primary root	Seedling stage [2–4 cm length]	1055	995	66	67	Baldauf et al. (2018)
		Seedling stage [6–8 cm length]	1073	1071	64	67	
		Seedling stage [10–12 cm length]	1046	901	65	71	
B73xW64A	Primary root	Seedling stage [2–4 cm length]	1061	928	61	66	Baldauf et al. (2018)
		Seedling stage [6–8 cm length]	1069	924	64	70	
		Seedling stage [10–12 cm length]	1060	899	65	73	
Oh43xW64A W64AxOh43	Primary root	Seedling stage [2–4 cm length]	941^3^	864^4^	57	64	Baldauf et al. (2018)

Among the different maize hybrids and tissues investigated in the different transcriptomic studies (Table 2), no uniform set of SPE genes was identified. However, among the highly genotype-specific SPE genes, evolutionary young non-syntenic genes were significantly enriched (Fig. 3F; Table 2; Paschold et al. 2014; Baldauf et al. 2018, 2022; Li et al. 2021b). The maize genome contains two evolutionarily distinct subsets of genes categorized relative to the presence of syntenic orthologs in the closely related sorghum genome (Schnable and Lyons 2011). Maize genes, which are syntenic to sorghum genes, are evolutionarily older than non-syntenic maize genes that have no sorghum ortholog. It was suggested that non-syntenic genes often contribute to the capacity of plants to adapt to fluctuating environmental conditions (Schnable 2015). Considering the ability of maize hybrids to cope better with fluctuating environmental conditions than parental inbred lines (Betrán et al. 2003), it was proposed that an enrichment of non-syntenic SPE genes could contribute to the adaptability of hybrids. This notion is supported by a study, which demonstrated that SPE genes display a very stable expression pattern under water deficit conditions (Marcon et al. 2017).

## Differential nutrient use efficiency of hybrids relative to inbred lines

The introduction of hybrid varieties, in combination with more efficient agricultural practices, including the application of synthetic fertilizers, resulted in remarkable yield increases (Fig. 2). Nitrogen (N) is an important nutrient that affects crop development. In the past thirty years, however, the rate of N fertilizer remained relatively stable but increases in grain yield are still noticeable (Fig. 2; Haegele et al. 2013; Einarsson et al. 2021). On the one hand, this can be attributed to the genetic gain obtained during germplasm selection for new hybrid varieties. On the other hand, this can be attributed to the increased ability of modern varieties to acquire and utilize soil nitrogen (Haegele et al. 2013; York et al. 2015; Emmett et al. 2018). Breeding hybrid varieties with high nutrient use efficiency is with respect to ecological and economic reasons an important target.

Roots are the main plant organ to absorb nutrients, thus an efficient root system is essential. In the past 100 years of maize breeding with the aboveground target of yield increase, genotypes with a more shallower and deeper root system with less nodal roots, but longer lateral roots were selected (York et al. 2015; Favela et al. 2021).

Hybrids are more efficient in the absorption of nutrients in comparison to inbred lines (Wei et al. 2018; Li et al. 2021a). The improved uptake of nutrient was positively correlated to grain yield and several root characteristics (Wei et al. 2018). Similarly, the hybrid generated from two contrasting Arabidopsis accessions, revealed superior phosphate acquisition efficiency (PAE) in comparison to its parents. The observed hybrid vigor was attributed to the accumulation of favorable dominant genes at several loci relating to PAE (Narang and Altmann 2001). A transcriptomic study revealed that a maize hybrid responded differently to N-limiting conditions than its parental inbred lines. Genes important for N metabolism were enriched in the hybrid while not in the parents, resembling in its expression pattern the higher nitrogen use efficient (NUE) parent (Bi et al. 2014). In several species, QTLs related to root system architecture and NUE have been identified and can be applied in marker-assistant selection for crop improvement (Feng et al. 2010; Li et al. 2015; Wang et al. 2017; Ma et al. 2020). Recently, several heterotic loci for diverse root traits have been identified displaying overdominant effects in hybrids under contrasting nitrogen levels (Xu et al. 2020).

## The impact of root microbes on hybrid vigor

The rhizosphere is the narrow region of soil of only a few millimeters that is directly influenced by root exudates and influences the root system via the associated microorganisms. These microorganisms are collectively designated as the root microbiome (Bai et al. 2022). The rhizosphere microbiome can influence root architecture and function (Durán et al. 2018) and plant development in general by facilitating the acquisition of mineral nutrients or by enhancing the resistance to biotic and abiotic stress (reviewed in Bai et al. 2022). It was demonstrated that during past decades of maize breeding under increased levels of nitrogen application, the selection of germplasm affected the microbial assembly in the rhizosphere of elite inbred lines (Favela et al. 2021), highlighting the importance of the rhizosphere microbiome for plant productivity. Microbes are attracted by organic exudates of plant roots (Sasse et al. 2018). These exudates vary depending on the genotype or root type and thus the microbial community (Wagner et al. 2020; Birt et al. 2022). The rhizosphere microbiomes associated with maize hybrids differed to those of the parental inbred lines grown under natural field conditions (Wagner et al. 2020). Furthermore, it was demonstrated that soil-borne microbes influence the performance of hybrids itself and robustly mediate heterosis in maize. Under sterile soil conditions, inbred lines performed as good as their hybrid offspring, but the effect of heterosis for root biomass could be restored after inoculating the plants with a simple community of bacteria (Wagner et al. 2021). The positive effect of soil microbes on plant development is supported by several studies (e.g., Kwak et al. 2018; Lu et al. 2018; Yu et al. 2021b). However, further research is necessary to disentangle the interaction between maize hybrids, including the underlying hybrid vigor, and their microbiome.

## Conclusion: applying molecular studies for trait improvement

A comprehensive understanding of the molecular principles of heterosis is the basis to accelerate crop improvement and to optimize breeding. Transcriptomic studies have been widely applied to investigate the gene expression landscape of hybrids. Employing transcriptomic data as biomarkers for predicting hybrid performance can efficiently increase the selection response to support breeding programs (Zenke-Philippi et al. 2017; Schrag et al. 2018). To this end, understanding heterosis manifestation early in development is highly beneficial for hybrid screening at high throughput (reviewed in Paschold et al. 2010). The phenomenon of heterosis is utilized mainly for traits related to increased yield or plant biomass. Since yield and biomass depend on nutrient and water availability, the belowground part of plants is an important target for crop improvement (Bishopp and Lynch 2015; Rogers and Benfey 2015; Hochholdinger 2016). Hybrids are more nutrient use efficient than their parental inbred lines (Wei et al. 2018; Li et al. 2021a) and soil microbes have positive effects on plant development (e.g., Kwak et al. 2018; Lu et al. 2018; Yu et al. 2021b). Therefore, understanding the mechanisms underlying nutrient absorption and utilization in hybrids and their interplay with their root microbiome is of utmost importance and the basis for implementing this knowledge to a more sustainable agriculture and to further unravel the phenomenon of heterosis.
